# Salidroside Stimulates Mitochondrial Biogenesis and Protects against H_2_O_2_-Induced Endothelial Dysfunction

**DOI:** 10.1155/2014/904834

**Published:** 2014-04-24

**Authors:** Shasha Xing, Xiaoyan Yang, Wenjing Li, Fang Bian, Dan Wu, Jiangyang Chi, Gao Xu, Yonghui Zhang, Si Jin

**Affiliations:** ^1^Department of Pharmacology, Tongji Medical College, Huazhong University of Science and Technology, Wuhan 430030, China; ^2^The Key Laboratory of Drug Target Research and Pharmacodynamic Evaluation of Hubei Province, Wuhan 430030, China

## Abstract

Salidroside (SAL) is an active component of *Rhodiola rosea* with documented antioxidative properties. The purpose of this study is to explore the mechanism of the protective effect of SAL on hydrogen peroxide- (H_2_O_2_-) induced endothelial dysfunction. Pretreatment of the human umbilical vein endothelial cells (HUVECs) with SAL significantly reduced the cytotoxicity brought by H_2_O_2_. Functional studies on the rat aortas found that SAL rescued the endothelium-dependent relaxation and reduced superoxide anion (O2^∙−^) production induced by H_2_O_2_. Meanwhile, SAL pretreatment inhibited H_2_O_2_-induced nitric oxide (NO) production. The underlying mechanisms involve the inhibition of H_2_O_2_-induced activation of endothelial nitric oxide synthase (eNOS), adenosine monophosphate-activated protein kinase (AMPK), and Akt, as well as the redox sensitive transcription factor, NF-kappa B (NF-**κ**B). SAL also increased mitochondrial mass and upregulated the mitochondrial biogenesis factors, peroxisome proliferator-activated receptor gamma-coactivator-1alpha (PGC-1**α**), and mitochondrial transcription factor A (TFAM) in the endothelial cells. H_2_O_2_-induced mitochondrial dysfunction, as demonstrated by reduced mitochondrial membrane potential (Δ**ψ**m) and ATP production, was rescued by SAL pretreatment. Taken together, these findings implicate that SAL could protect endothelium against H_2_O_2_-induced injury via promoting mitochondrial biogenesis and function, thus preventing the overactivation of oxidative stress-related downstream signaling pathways.

## 1. Introduction

The role of oxidative stress in the development of the endothelial dysfunction has been studied extensively [1–4]. Excessive reactive oxygen species (ROS) not only reduces bioavailable nitric oxide (NO) through direct reaction to form peroxynitrite, but also leads to eNOS uncoupling and further induces more ROS production [5].

As a major cellular source of ROS, the contributions of mitochondria to the detrimental effects of cardiovascular risk factors have recently received increased attention [6–8]. Excessive mitochondrial ROS (mtROS) act to inspire pathologic cell-signaling cascades under conditions of comprehensive excessive oxidative stress [9–11]. NF-kappa B (NF-*κ*B) activation occurs secondary to excessive mitochondrial ROS production in the endothelium, participating in a range of proinflammatory and prothrombotic alterations in the endothelial cells [12, 13].

Endothelial mitochondria have been found to have crucial roles in vascular path-physiology [14–16], and increasing evidences have indicated the importance of mitochondrial dysfunction in various vascular diseases, such as atherosclerosis, heart failure, and cardiac ischemia/reperfusion injury [17–19]. Previous studies have shown that dysregulation of mitochondrial biogenesis represents an early manifestation of endothelial dysfunction, shifting cell metabolism toward metabolic hypoxia in animals with impaired NO bioavailability [14]. Impairment of mitochondrial biogenesis is frequently observed in atherosclerosis and is thus likely to contribute to cellular energetic imbalance, oxidative stress, and endothelial dysfunction in these pathological conditions [20]. Since increased mitochondrial production of ROS due to impaired mitochondrial biogenesis also appears to be a key event in the development of aging-related vascular pathologies [13, 21, 22], identification of mechanisms that promote mitochondrial biogenesis in the endothelial cells may provide new clues on the pathogenesis of vascular disease.

The health of mitochondria is in part regulated by their biogenesis and peroxisome proliferator-activated receptor gamma-coactivator-alpha (PGC-1*α*) is regarded as the key regulator [23]. In endothelial cells, PGC-1*α* also orchestrates cellular defenses against oxidative stress [24]. Mitochondrial transcription factor A (TFAM) is responsible for the transcriptional control of mtDNA and its translocation to the mitochondria is important to initiate mtDNA transcription and replication [25].

As described above, mitochondria are highly dynamic organelles, and their biogenesis is likely to be involved in the regulation of endothelial cell metabolism, redox regulation, and signal transduction [6, 16, 26]. Pathways that regulate mitochondrial biogenesis are potential therapeutic targets for the amelioration of endothelial dysfunction and vascular diseases [27].

Salidroside (SAL) is an active ingredient of the root of* Rhodiola rosea*, a well-known herb used to relieve high altitude sickness [28]. SAL has also been used to enhance both the physical and mental performance. SAL upregulates the levels of antioxidative enzymes glutathione peroxidase-1 and thioredoxin-1 to counteract oxidative stress [29]. Previous studies have shown that SAL promotes DNA repair enzyme Parp-1 to counteract oxidative stress [30]. Meanwhile, a recent study reported that SAL attenuated homocysteine-induced endothelial dysfunction by reducing oxidative stress [31]. As noted above, there is tightly relationship between reduction of mitochondrial biogenesis and endothelial dysfunction; the present study was conducted to determine whether SAL recovers the endothelial dysfunction induced by H_2_O_2_ through stimulating mitochondrial biogenesis and counteracting the oxidative stress-related eNOS and NF-*κ*B signaling pathways.

## 2. Materials and Methods

### 2.1. Animals

Animals were treated in accordance with the guide for the Care and Use of Laboratory Animals published by the US National Institutes of Health and approved by the Local Animal Care Committee. Healthy Wistar rats (200–250 g) were purchased from the Center of Experimental Animals (Tongji Medical College, Huazhong University of Science and Technology, China) and maintained in a controlled environment with a light/dark cycle of 12 h, a temperature of 20 ± 2°C, and humidity of 50 ± 2%.

### 2.2. Cell Culture

The collection of human umbilical cords was approved by the Ethics Committee of Tongji Medical College, Huazhong University of Science and Technology (Wuhan, China) and conducted in accordance with the Declaration of [32]. Primary cultured human umbilical vein endothelial cells (HUVECs) were prepared as described in [33]. In brief, the umbilical cord was washed with cold PBS and then infused with 0.25% trypsin. After digestion stopped, the cells were collected by centrifuging for 10 min at 1 000 rpm. The cells were resuspended and then cultured in endothelial cell medium (ECM, Sciencell, Carlsbad, CA) at 37°C in an incubator with a humidified atmosphere of 5% CO_2_. In all experiments, cells were used at passages 2–7.

### 2.3. Cell Viability Assays

To study the effect of SAL on H_2_O_2_-induced cytotoxicity, HUVECs were inoculated at a density of 2 × 10^4^ per well in 96-well plates and cultured overnight; the cell viability was evaluated using the Cell Counting Kit-8 (Dojindo Laboratories, Kumamoto) [34]. Briefly, the HUVECs treated with H_2_O_2_ (Sigma, 4 h) or SAL (National Institute for Food and Drug Control, purity > 98%, 24 h) at the indicated concentration in OPTI-MEM (Gibco) before cell viability was measured. Moreover, to study the effect of SAL on H_2_O_2_-induced inhibition of cell viability, the cells were cultured as described above; after treatment with SAL for 20 h, the H_2_O_2_ (100 *μ*M) was added to the medium for another 4 hours. At the end of the time period, the culture medium was removed. The cells were washed twice with PBS and incubated with CCK-8 solution at 37°C for 30 min. The absorbance was measured using a microplate reader with a test wavelength of 450 nm.

### 2.4. Free Radical Measurement in Cell Free System

The effects of SAL on scavenging hydroxyl radical (OH^∙^), superoxide radical (O_2_^−^), and H_2_O_2_ were measured with commercially available kits (Nanjing Jiancheng Bioengineering Institute, China) according to the manufacturer's instructions. In brief, the OH^∙^ was generated by the Fenton reaction and then treated with a chromogenic substrate nitrotetrazolium blue chloride (NBT) to yield a stable colored substance, which was measured using a microplate reader with a test wavelength of 450 nm. O_2_^−^ was generated by the xanthine/xanthine oxidase system, O_2_^−^ was detected by nitrite method, and the absorbance at 550 nm was measured. The reaction product of H_2_O_2_ and molybdic acid can be detected at 405 nm. Deionized water and ascorbic acid (Vc) were used as the blank and positive controls, respectively. The inhibition rate = (optical density of blank control groups—optical density of treatment groups)/optical density of blank control groups.

### 2.5. Vascular Function Measurement

The thoracic segments of the rat aorta were dissected and the surrounding connective tissues were cleaned off. Each aorta was cut into ring segments of 2~3 mm in length. The aortic rings were mounted in organ chambers filled with Krebs-Henseleit (KH) solution at 37°C with constant bubbling of 95% oxygen/5% carbon dioxide. KH solution contained (mM) 133 NaCl, 4.75 KCl, 1.5 CaCl_2_, 1.25 MgCl_2_, 25 NaHCO_3_, and 11 D-glucose. Isometric tension was recorded with a force transducer (RM6240C, Chengdu Instrument Factory). A basal tension of 20 mN was applied to each vascular ring. After being placed in organ baths for 90 min, 0.5 *μ*M phenylephrine (PE, Sigma) was first administered to the rings to test their contractility and then 1 *μ*M acetylcholine (ACh, Sigma) was administered to assess the integrity of the endothelial layer. Rings with less than 80% relaxation response to ACh were discarded. The aortic rings were pretreated with or without 10 *μ*M SAL for 30 min before H_2_O_2_ 100 *μ*M was added to the bath. After precontracting with PE (0.1 *μ*M), ACh (1 × 10^−8^~1 × 10^−4 ^M) was added cumulatively to the bath to evoke the endothelium-dependent relaxation. The relaxation rate is the ratio between the tension relaxed by ACh and the tension contracted by PE.

### 2.6. Measurement of NO and Superoxide Anion (O_2_^∙−^)

To measure intracellular NO and O_2_^∙−^ levels, the NO and O_2_^∙−^-specific fluorescent dye 4,5-diaminofluorescein diacetate (DAF-FM-DA, Beyotime Institute of Biotechnology) [35] and dihydroethidium (DHE, Beyotime Institute of Biotechnology) [36] were used to measure intracellular NO and O_2_^∙−^ levels, respectively. Briefly, confluent HUVECs in 96-well plates, after SAL (10 *μ*M) or PBS treatment for 24 h, were washed twice with PBS followed by staining with 2.5 *μ*M DAF-FM-DA or 5 *μ*M DHE for 30 min at 37°C. After washing twice with PBS, the fluorescence intensities were measured as basal, and then 100 *μ*M H_2_O_2_ was added. Using a fluorescence spectrophotometer (TECAN, INFINITE F200PRO), the fluorescence intensities of DAF-FM-DA were measured at an excitation wavelength of 485 nm and an emission wavelength of 535 nm, respectively, and DHE were measured at an excitation wavelength of 535 nm and an emission wavelength of 610 nm, respectively.

### 2.7. NF-*κ*B Activity Assay

An ELISA-based assay was used to measure the NF-*κ*B activity, as described previously [37–39]. In brief, after stimulation, cells were rinsed twice with cold PBS, and then RIPA containing a protease inhibitor cocktail (Roche, Basel, Switzerland) was added. After incubation on ice for 30 min, the lysate was centrifuged for 15 min at 14 000 rpm and the supernatant was collected. After being quantified with BCA reagent (Pierce, Rockford, IL), the cell extracts were incubated in a 96-well plate coated with the oligonucleotide containing the NF-*κ*B consensus-binding site (5′-GGGACTTTCC-3′). Activated transcription factors from extracts specifically bound to the respective immobilized oligonucleotide. NF-*κ*B activity was then detected with the primary antibody to NF-*κ*B p65 (1 : 1000, Proteintech, China) and secondary antibody conjugated to horseradish peroxidase (1 : 10 1000, Abbkine, CA). Tetramethylbenzidine (100 *μ*L, Sigma) was added in each microwell at 37°C before adding 100 *μ*L of stopping solution (2M H_2_SO_4_). NF-*κ*B activity was finally determined as absorbance values measured with a microplate reader at a wavelength of 450 nm.

### 2.8. Measurement of Mitochondrial Mass

Mitochondria mass was determined by using MitoTracker green, a mitochondrial-selective membrane potential-independent dye [40]. The cells grown on cover slips coated with 2% gelatin were incubated with SAL (1, 10 *μ*M) for 24 h. At the end of the incubation, suspensions were removed and the cells were incubated with 200 nM MitoTracker green (Beyotime Institute of Biotechnology, China) in 37°C for 30 min. The images were captured with a fluorescence microscope (Olympus FV500) using 40× magnification objective [41]. The integrated fluorescence intensities were measured using the Image-Pro Plus software and normalized to the number of cells.

### 2.9. Measurement of Intracellular ATP Levels

After treatment, the HUVECs from each well of a 6-well dish (5 × 10^5^/well) were washed twice with cold PBS and lysed with 0.5 M perchloric acid and briefly sonicated (5 to 10 times of a 1 sec burst) until cells were clearly disrupted. Samples were then neutralized with 2 M KOH and then centrifuged to remove the precipitate. ATP content was analyzed by HPLC (Agilent, Palo Alto, CA) with an LC-18T reverse-phase column (Agilent, Palo Alto, CA) at a flow rate of 0.3 mL/min, and the absorbance at 254 nm was recorded. The elution peak was compared with ATP standards (National Institute for Food and Drug Control, Beijing) to confirm its identity.

### 2.10. Assessment of Mitochondrial Membrane Potential (Δ*ψ*m)

JC-1 is a positively charged fluorescent compound which is taken up by mitochondria proportionally to the inner mitochondrial membrane potential [42]. The ratio of red (J-aggregate)/green (monomeric JC-1) emission is directly proportional to the Δ*ψ*m. HUVECs were grown on 96-well plates treated with SAL (10 *μ*M) for 20 h, and then H_2_O_2_ (100 *μ*M) was added for another 4 h. Cells were rinsed with PBS and incubated in 100 *μ*L JC-1 staining solution at 37°C for 20 min. Cells were then rinsed twice with JC-1 washing solution and analyzed with a fluorescence spectrophotometer. J-aggregates were recorded with an excitation wavelength of 535 nm and an emission wavelength of 610 nm, respectively, and monomeric JC-1 was recorded with an excitation wavelength of 485 nm and an emission wavelength of 535 nm, respectively.

### 2.11. Western Blot Analysis

Cells were homogenized in ice-cold RIPA lysis buffer containing protease inhibitor cocktail and phosSTOP (Roche, Basel, Switzerland). Equal amounts of protein (60 *μ*g) were mixed with the loading buffer (Beyotime Institute of Biotechnology), boiled for 10 min, and separated by SDS-PAGE. After electrophoresis, proteins were transferred to polyvinylidene difluoride membranes (PVDF, Millipore, Temecula, CA). The membranes were blocked for 1 h in 5% milk. The membranes were then incubated overnight at 4°C with one of the following specific primary antibodies: rabbit anti-eNOS ser1177, anti-eNOS, anti-AMPK*α* thr172, anti-AMPK*α*, anti-*β*-actin (1 : 1 000, Cell Signaling Technology, Beverly, MA), anti-Akt ser473 (1 : 1 000, EPITOMICS, CA), anti-Akt (1 : 600, Proteintech), anti-TFAM (1 : 300, Proteintech), and anti-PGC-1*α* (1 : 200, Santa Cruz, CA). After washing, the membranes were incubated for 2 h at room temperature with secondary antibodies (Goat anti-rabbit IgG, goat anti-mouse IgG, 1 : 10 000, Abbkine, CA) and then washed. Finally, the blots were developed with enhanced chemiluminescence detection reagents (Thermo Scientific, Waltham, MA). Membranes were scanned using the MicroChemi bioimage analyzer (NDR, Israel) and quantified using Image J program and normalized against *β*-actin.

### 2.12. Statistical Analysis

All data in this study are expressed as the mean ± SEM from at least three separate experiments. SPSS 13.0 was used for statistical analysis. Individual group statistical comparisons were analysed by unpaired Student's *t*-test with Bonferroni correction, and multiple groups comparisons were evaluated by one-way ANOVA with post hoc testing. A probability value of *P* < 0.05 was considered statistically significant.

## 3. Results

### 3.1. SAL Alleviates the Cytotoxicity Induced by H_2_O_2_ in HUVECs

**Figure 1:**
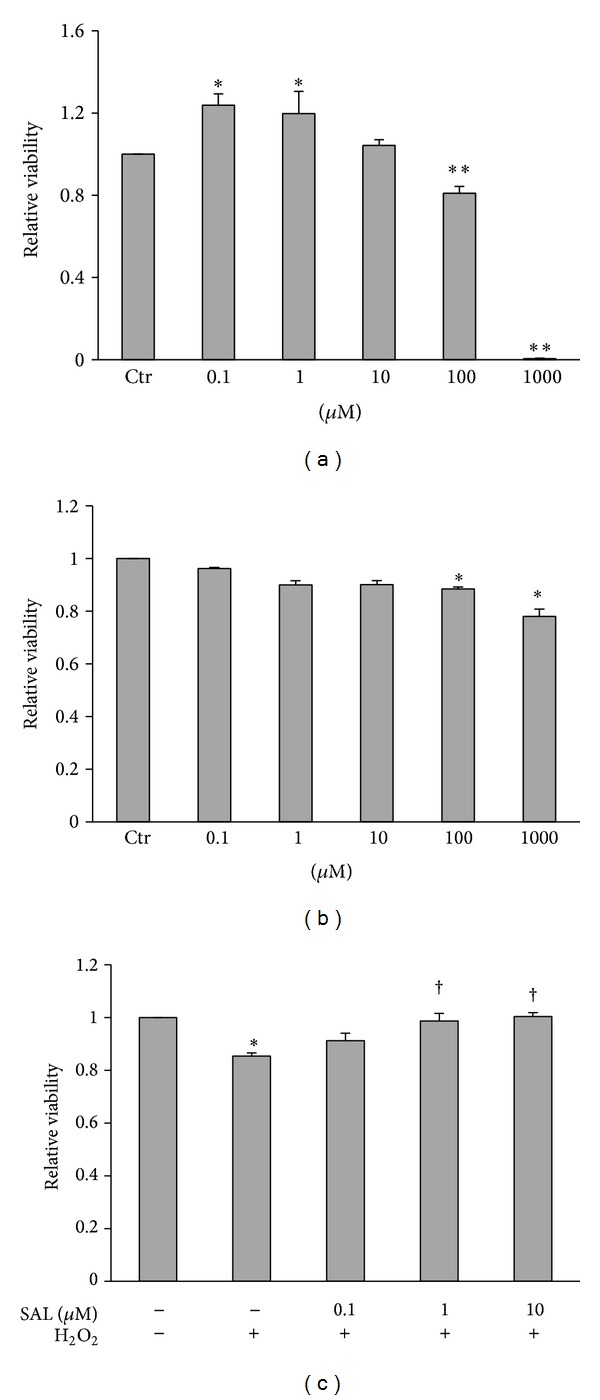
Protective effects of SAL on H_2_O_2_-induced cytotoxicity in HUVECs. (a) HUVECs were exposed to various concentrations of H_2_O_2_ for 4 h. (b) Cells were exposed to various concentrations of SAL for 24 h. (c) HUVECs were pretreated with SAL or vehicle for 20 h and then exposed to H_2_O_2 _(100 *μ*M) for 4 h. Cell viability was detected by CCK-8. Cell viability in untreated cells was assigned the value of 1. *P < 0.05, **P < 0.01 versus control; ^†^ *P* < 0.05 versus H_2_O_2_, *n* = 3–5.

In this study, H_2_O_2_ was used to induce oxidative stress in HUVECs. After exposure to H_2_O_2_ (100–1000 *μ*M) for 4 h, HUVECs viability was reduced (Figure 1(a)). SAL at the concentration below 10 *μ*M had no obvious effect on cell viability compared to control (Figure 1(b)). Pretreatment with SAL could reduce cell death induced by H_2_O_2_ in a concentration-dependent manner (Figure 1(c)).

### 3.2. Effect of SAL on ROS in Cell Free System

**Figure 2:**
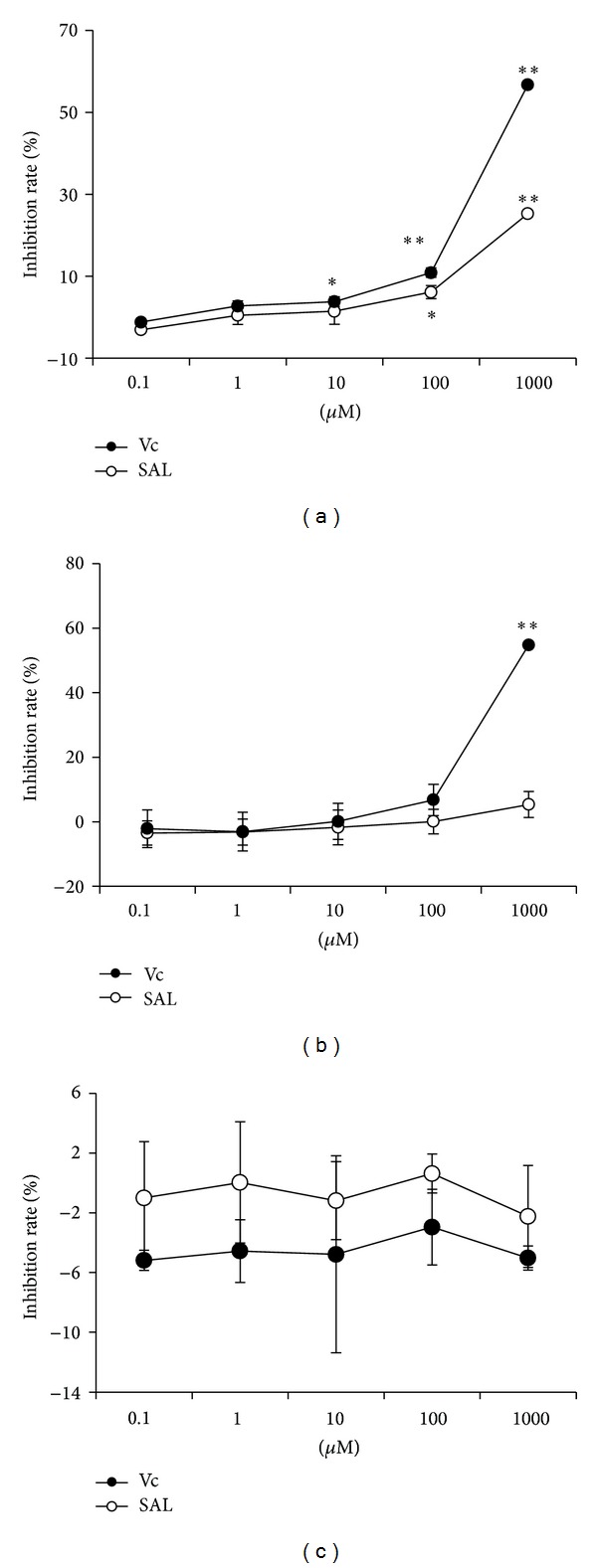
Effect of SAL on cell free ROS detection using different assay systems. (a) OH^∙^ was generated by Fenton reaction, (b) O_2_^−^ was generated by xanthine/xanthine oxidase, and (c) H_2_O_2_ was exogenously added. *P < 0.05, **P < 0.01 versus blank control, *n* = 3.

SAL has the effect of scavenging OH^∙^ but not O_2_^−^ or H_2_O_2_ at indicated concentration (Figures 2(a), 2(b), and 2(c)).

### 3.3. SAL Recovers H_2_O_2_-Induced Impairment of Endothelium-Dependent Relaxation in Rat Aortas

**Figure 3:**
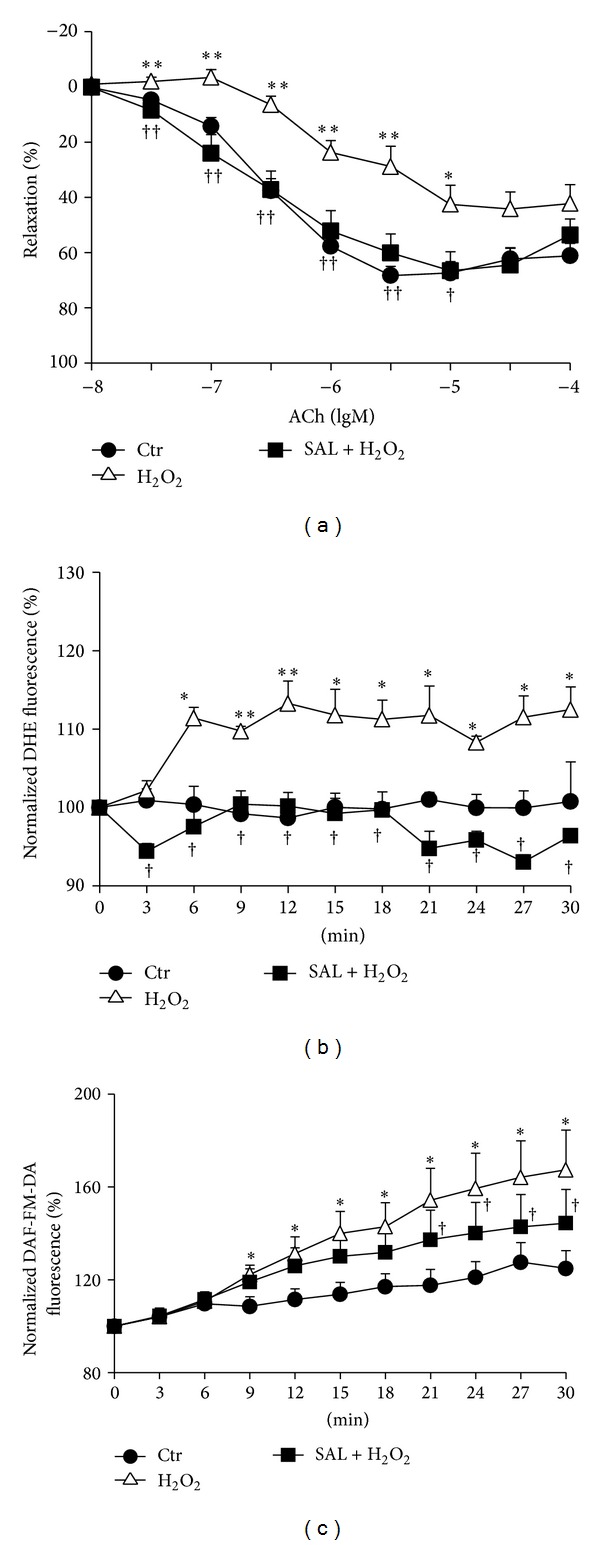
Protective effects of SAL on H_2_O_2_-induced impairment of endothelium-dependent relaxation. (a) Rat thoracic aorta was pretreated with or without SAL for 30 min, and then H_2_O_2_ (100 *μ*M) was added to incubate for another 30 min. After precontracting with 1 *μ*M PE, ACh was added accumulatively. Complete relaxation of aorta induced by ACh was considered as 100%. *P < 0.05, **P < 0.01 versus control; ^†^ *P* < 0.05, ^††^ *P* < 0.01 versus H_2_O_2_, *n* = 8–16. (b) HUVECs were pretreated with or without 10 *μ*M SAL for 20 h and then loaded with DHE dye. After acquisition of basal data, H_2_O_2_ (100 *μ*M) was added and the fluorescence was measured every 5 min within 30 min. The fluorescence of basal data was assigned the value of 100%. *P < 0.05, **P < 0.01 versus control; ^†^ *P* < 0.05 versus H_2_O_2_, *n* = 4. (c) HUVECs were pretreated with or without SAL for 20 h and then loaded with DAF-FM-DA dye. After acquisition of basal data, H_2_O_2_ (100 *μ*M) was added and the fluorescence was measured every 5 min within 30 min. The fluorescence of basal data was assigned the value of 100%. *P < 0.05 versus control; ^†^ *P* < 0.05 versus H_2_O_2_, *n* = 4.

Treatment with H_2_O_2_ (100 *μ*M) for 30 min markedly attenuated ACh-induced endothelium-dependent relaxation (EDR) in rat aortas. Exposure to SAL (10 *μ*M, 30 min) prior to the addition of H_2_O_2_ partially rescued the impaired EDR (Figure 3(a)). Meanwhile, pretreatment with SAL (10 *μ*M, 24 h) inhibited NO and O_2_^∙−^ production induced by H_2_O_2_ (100 *μ*M) (Figures 3(b) and 3(c)).

### 3.4. SAL Decreased eNOS Activation Induced by H_2_O_2_ in HUVECs

**Figure 4:**
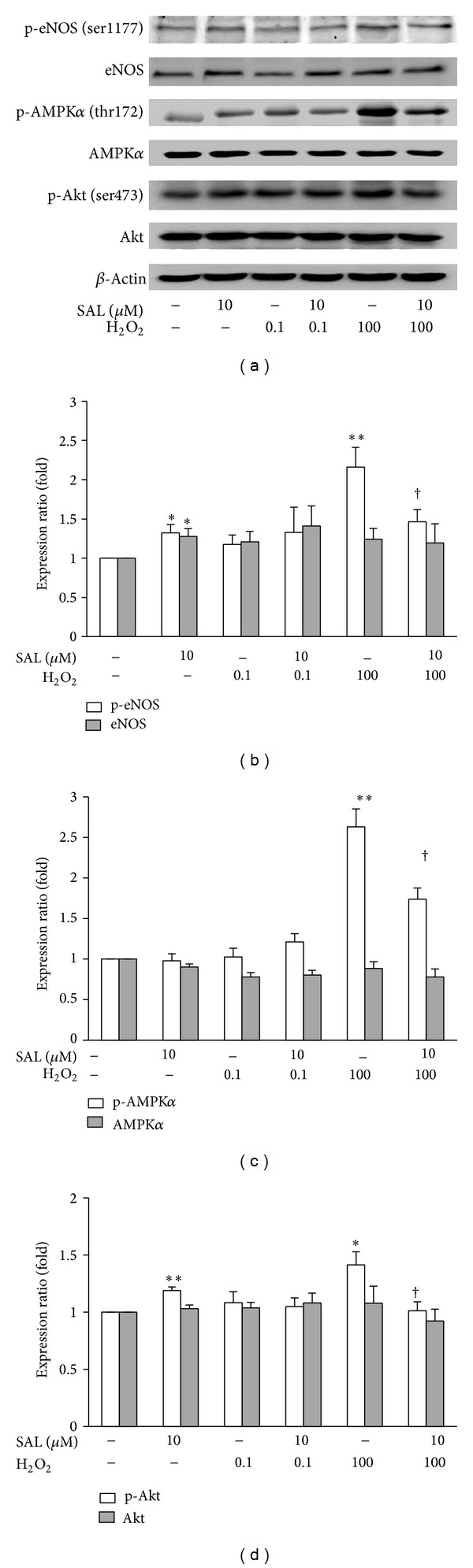
Effects of SAL on H_2_O_2_-induced activation of eNOS, AMPK, and Akt. After pretreatment with SAL (10 *μ*M) for 20 h, H_2_O_2_ (0.1 *μ*M or 100 *μ*M) was added for another 4 h, and the lysates were analyzed by western blot. (a) Representative immunoblots. (b, c, d) The histogram shows quantitation of eNOS-ser1177, eNOS, AMPKa-thr172, AMPK, Akt-ser473, and Akt expression. The expressions were normalized to that obtained in untreated cells. *P < 0.05, **P < 0.01 versus control; ^†^ *P* < 0.05 versus H_2_O_2_, *n* = 4–6.

Compared with control, H_2_O_2_ (100 *μ*M, 4 h) or SAL (10 *μ*M, 24 h) treatment significantly increased eNOS phosphorylation at ser1177 and Akt phosphorylation at Ser473 in HUVECs. Pretreatment with SAL (10 *μ*M) for 24 h attenuated the activation of eNOS, AMPK*α*, and Akt induced by 100 *μ*M H_2_O_2_ (Figures 4(a), 4(b), 4(c), and 4(d)).

### 3.5. SAL Inhibited the Activation of Transcription Factor NF-*κ*B Induced by H_2_O_2_ in HUVECs

**Figure 5:**
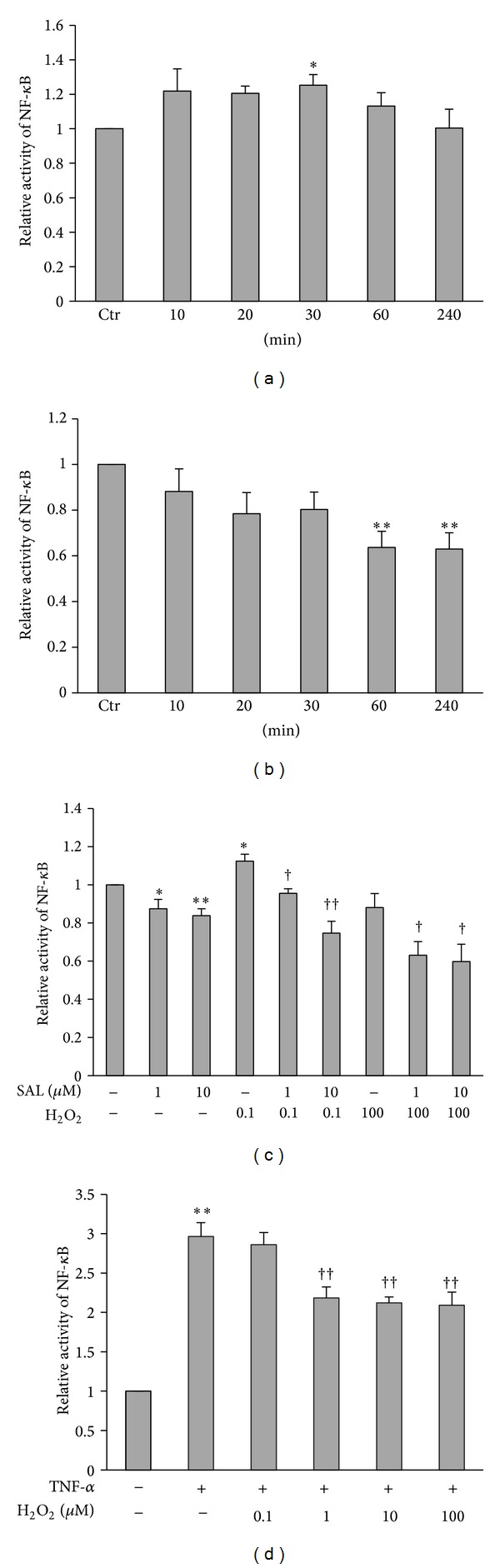
Effects of SAL on H_2_O_2_-induced activation of transcription factor NF-*κ*B. Effects of H_2_O_2_ (0.1 *μ*M) (a) and SAL (10 *μ*M) (b) on transcription factor NF-*κ*B activity. H_2_O_2_ (0.1 *μ*M) or SAL (10 *μ*M) was added for the indicated periods of time (0–4 h). The transcription activity in untreated cells was assigned the value of 1. *P < 0.05, **P < 0.01 versus control, *n* = 4. (c) Effects of SAL on H_2_O_2_-induced NF-*κ*B activity. HUVECs were pretreated with SAL (1, 10 *μ*M) for 10 min and then incubated with H_2_O_2 _(0.1, 100 *μ*M) for 30 min. *P < 0.05, **P < 0.01 versus control; ^†^ *P* < 0.05, ^††^ *P* < 0.01 versus H_2_O_2_, *n* = 4–6. (d) HUVECs were treated with TNF-*α* (30 ng/mL) for 30 min, and then total protein was extracted. The proteins were incubated with increasing concentrations of H_2_O_2 _(0.1, 1, 10, and 100 *μ*M) for 30 min, and then the activities of NF-*κ*B were detected as described in Section 2. The transcription activity was normalized to that obtained in untreated cells. **P < 0.01 versus control, ^††^ *P* < 0.01 versus TNF-*α*, *n* = 3.

Activation of the transcription factor NF-*κ*B has been associated with endothelial cells dysfunction and vascular inflammation in atherogenesis [43]. In line with previous studies, exposure to H_2_O_2_ could result in transient activation of NF-*κ*B (Figure 5(a)), while exposure to SAL (10 *μ*M) for indicated time significantly decreased the activity of transcription factor NF-*κ*B in a time-dependent manner (Figure 5(b)). Pretreatment with SAL inhibited the activation of the NF-*κ*B induced by 0.1 *μ*M H_2_O_2_ (Figure 5(c)). To our surprise, we found that exposure to H_2_O_2_ (0.1 *μ*M) for 30 min could result in transient activation of NF-*κ*B, whereas H_2_O_2_ at the concentration of 100 *μ*M slightly reduced rather than increased the NF-*κ*B activity. SAL pretreatment can further reduce the activity of NF-*κ*B (Figure 5(c)). To explain this result, we used TNF-*α*, a classical NF-*κ*B inducer to treat HUVECs to detect the direct effect of H_2_O_2_ on DNA binding activity. The result indicated that H_2_O_2_ inhibited the DNA binding activity of activated NF-*κ*B in a dose-dependent manner (Figure 5(d)).

### 3.6. SAL Induced Mitochondrial Biogenesis in Endothelial Cells

**Figure 6:**
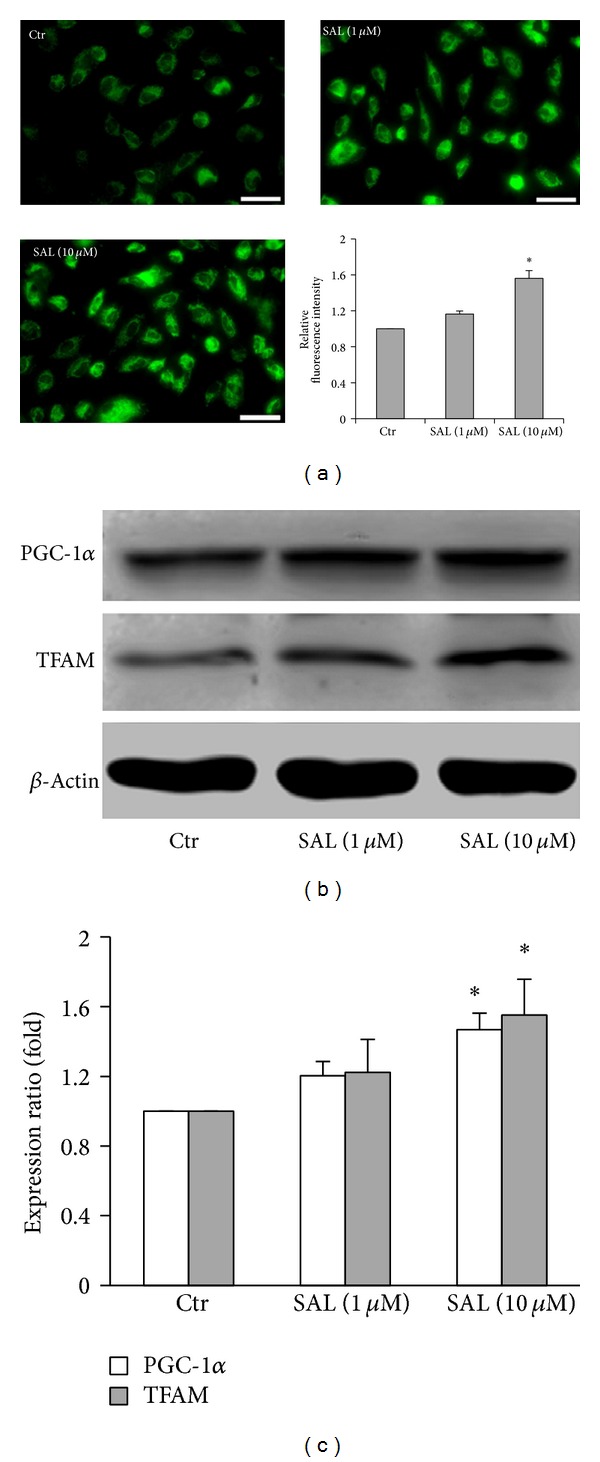
Effects of SAL on mitochondrial biogenesis. (a) Mitochondria mass was quantified using Mito Tracker Green. Scale bars = 50 *μ*m. The fluorescence in untreated cells was assigned the value of 1. *P < 0.05 versus control, *n* = 4. HUVECs were treated with SAL (1, 10 *μ*M) for 24 h, and the total protein was extracted; the lysates were analyzed by western blot. (b) Representative immunoblots. (c) Summary histograms of the relative density of PGC-1*α* and TFAM normalized to *β*-actin. The expression in untreated cells was assigned the value of 1. *P < 0.05 versus control, *n* = 5.

Our results showed that SAL (10 *μ*M) increased the fluorescent intensity of MitoTracker green, suggesting that SAL increased the mitochondrial mass in HUVECs (Figure 6(a)). Consistent with this, the expression of PCG-1*α* and TFAM, the key regulators of mitochondrial biogenesis were significantly increased in HUVECs incubated with SAL (Figures 6(b) and 6(c)).

### 3.7. SAL Restores H_2_O_2_-Induced Mitochondrial Dysfunction

**Figure 7:**
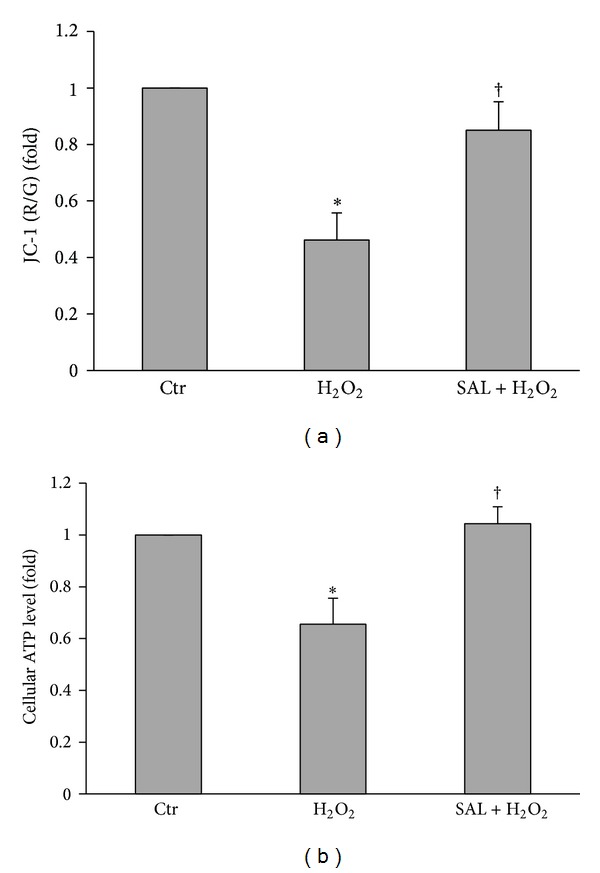
Effects of SAL on H_2_O_2_-induced mitochondrial dysfunction. HUVECs were pretreated with SAL (10 *μ*M) for 20 h, and then H_2_O_2 _(100 *μ*M) was added to incubate for another 4 h. (a) Mitochondrial membrane potential and (b) ATP content were detected as described in Section 2. ΔΨm and ATP content in untreated cells were assigned the value of 1. *P < 0.05 versus control; ^†^ *P* < 0.05 versus H_2_O_2_, *n* = 4.

We used independent parameters to evaluate mitochondrial function ATP production and ΔΨm. As shown in Figure 7, H_2_O_2_ (100 *μ*M) induced ΔΨm collapse (a) and decreased ATP production (b) after 4 h of treatment in cultured HUVECs. Pretreatment with SAL (10 *μ*M) for 24 h rescued mitochondrial function.

## 4. Discussion

There is increasing attention on the relationship between oxidative stress and endothelial cell injury [44–46]. The present study employed H_2_O_2_-induced oxidative stress in HUVECs as a cellular model to study the protective effect of SAL. In our experiments, pretreatment of SAL significantly prevents the impaired viability of HUVECs caused by H_2_O_2_ exposure. These results are in accordance with previous studies [47] and further confirmed the protective effects of SAL against injury induced by H_2_O_2_. Moreover, the antioxidative mechanism of SAL is not due to the direct reaction between SAL and H_2_O_2_ (Figure 2).

Overproduced ROS are known to harm the normal vascular function by limiting the beneficial effects of endothelium derived NO [48]. The enhanced production and release of ROS and/or the diminished bioavailability of NO within vascular wall lead to endothelial dysfunction that is widely believed to be the early key event in the pathogenesis of various vascular complications [4, 49]. Although H_2_O_2_ elicits relaxation in rat, mouse, and rabbit aortas [50–53] and stimulates eNOS, resulting in higher NO levels [54], the effect of prolonged elevation of H_2_O_2_ is to impair endothelium-dependent relaxation [55]. Previous study showed that SAL prevented homocysteine-induced endothelial dysfunction through curtailing oxidative stress [31]. In our study, pretreatment with H_2_O_2_ induced significant impairment of endothelial dependent relaxation, while SAL had the capacity to rescue this impairment. As expected, incubation of HUVECs with H_2_O_2_ strikingly increased intracellular O_2_^∙−^, and this can be suppressed by pretreatment with SAL. These results strongly suggest that SAL inhibits H_2_O_2_-induced ROS production, contributing to the restoration of the endothelium-dependent vasorelaxation.

NO, derived from the action of eNOS in endothelial cells, is one of the most important mediators in the regulation of endothelial functions [56]. Phosphorylation of eNOS at ser1177 activates eNOS, while increased oxidative flux directly scavenges NO to lower NO bioavailability [57] and subsequently impairs endothelium-dependent vasodilatation [58]. Interestingly, as shown in many previous studies, H_2_O_2_ directly upregulated the levels of NO in endothelial cells, suggesting that overproduction of NO by endothelial cells in response to H_2_O_2_ stimulation was intended to protect the cells, rather than damage cells [59, 60]. An intriguing question that arises from this study is why SAL tends to inhibit H_2_O_2_-induced eNOS activation and NO production, while it* per se* appears to upregulate eNOS expression and activation (Figures 4 and 3(c)). We speculated here that the upregulation of eNOS activity and NO production by long-term SAL treatment may be initiated by a transient mild mitochondrial depolarization and reduced oxygen demand, which in turn increase the tolerance of affected cells to subsequent oxidative insult that is greater in severity. But this needs to be further proved.

There are evidences that NO can repress the activation of NF-*κ*B through degradation of I*κ*B*α* [61] or inhibition of NF-*κ*B DNA binding [62]. We also analyzed the effect of SAL on the activation of NF-*κ*B induced by H_2_O_2_. Our date showed that SAL decreased the basal activity of NF-*κ*B, meanwhile blocking the activation of NF-*κ*B induced by H_2_O_2_ (Figure 5). H_2_O_2_ at the concentration of 100 *μ*M slightly reduced rather than increased the NF-*κ*B activity; meanwhile, pretreatment with SAL further reduced the activity. The possible explanation is that H_2_O_2_ *per se* induces the formation of active dimmers of P65 and P50 subunits and leads to their translocation to the nucleus. However, within the nucleus, the Cys 62 residue on the P50 subunit is oxidized to sulfenic acid and is further followed by S-glutathionylation, which inhibits the binding of NF-*κ*B to the DNA [63–65]. To strengthen this hypothesis, we detected the direct effect of H_2_O_2_ on DNA binding activity. We used TNF-*α*, a classical NF-*κ*B inducer to treat HUVECs. After cell lysis and total protein extraction, we incubated the proteins with increasing concentrations of H_2_O_2_ (0.1, 1, 10, and 100 *μ*M) for 30 min, and then the activities of NF-*κ*B were detected as described in Section 2. The results indicated that H_2_O_2_ dose dependently inhibited the DNA binding activity of activated NF-*κ*B. Since NF-*κ*B is a redox sensitive transcription factor, its activation participates in inflammation and mitochondrial biogenesis impairment [66]. We speculated that the protective effect of SAL on HUVECs might be related to an interference with the NF-*κ*B signaling pathway.

Moderate increases in NO stimulate mitochondrial biogenesis, mainly through cGMP-dependent gene expression and activation of regulatory factors including PGC-1*α* and TFAM [67, 68]. In cardiomyocytes, coimmunoprecipitation experiments demonstrated that the p65 subunit of NF-*κ*B is constitutively bound to PGC-1*α* coactivator and blocks its activation of gene transcription and that NF-*κ*B activation increases this binding [69]. Moreover, PGC-1*α* overexpression inhibits NF-*κ*B activation in human aortic smooth muscular and endothelial cells [70]. So it is reasonable that the agents stimulating PGC-1*α* expression and mitochondrial biogenesis in the endothelial cells are beneficial to prevent the development of cardiovascular disease. Here we reported for the first time that SAL increased mitochondrial mass in HUVECs (Figure 6(a)). Inducible mitochondrial biogenesis is very important in vascular health [27, 71]. Moreover, mitochondrial proliferation reduces the flow of electron per unit mitochondria; SAL-induced mitochondrial biogenesis may contribute to the reduction of mitochondrial ROS production in HUVECs.

To determine whether the effect of SAL on mitochondrial biogenesis is a consequence of the activation of mitochondrial biogenesis regulatory factors, we examined the expression of PGC-1*α* and TFAM. We found that SAL increased the expression of PGC-1*α* and TFAM (Figures 6(b) and 6(c)).

In addition to stimulating mitochondrial biogenesis, PGC-1*α* also contributes to the induction of ROS detoxifying enzymes, including catalase, superoxide dismutase, and heme oxygenase [72–75]. These findings imply that PGC-1*α*-mediated mitochondrial biogenesis in oxidative injured cells seems to offer a good source of “healthy mitochondria” which detoxify mtROS by a large antioxidant defense system containing numerous redox enzymes of the electron-transport chain, ultimately decreasing net ROS production. These effects of SAL on mitochondrial biogenesis may partially explain its antioxidant properties.

Many evidences have revealed that H_2_O_2_ caused endothelial cell injury by inducing mitochondrial dysfunction [76, 77]. Due to localization to the inner mitochondrial membrane, lack of histone-like coverage and a less efficient DNA repair system compared with nuclear DNA, mtDNA is prone to oxidative stress [78]. Furthermore, the mutation is more likely to affect gene integrity because of the absence of intron in the mitochondrial genome [79]. Damaged mitochondria produce less ATP but more greater amounts of ROS, potentiating the signal and entering a vicious circle, which aggravate cardiovascular diseases [80]. As shown in Figure 7, SAL pretreatment abrogated the H_2_O_2_-induced collapse of ΔΨm and rescued the mitochondrial function, proved by increased ATP production. These effects of SAL decrease the potential for mtDNA damaged by H_2_O_2_; moreover, SAL enhanced mitochondrial biogenesis, which provides healthy mitochondria to replace the mitochondrial components damaged by ROS and maintain normal mitochondrial function. The protective effect of SAL on mitochondria is of paramount importance to maintain the endothelial homeostasis.

**Figure 8:**
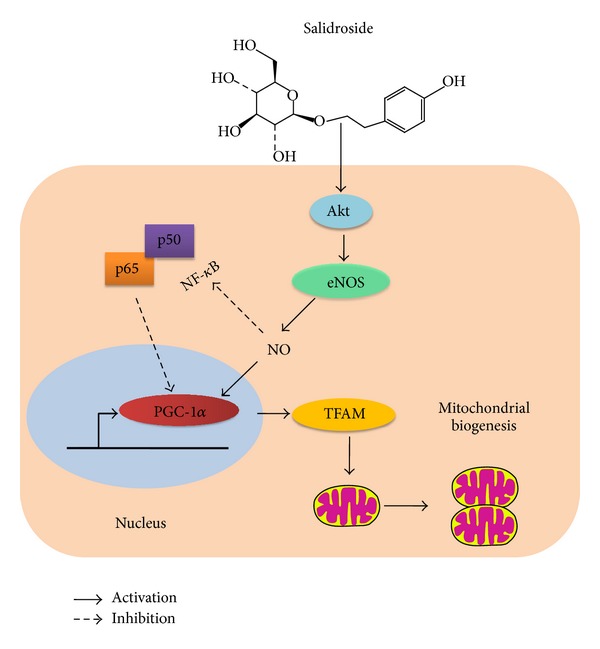
Schematic diagram of the potential mechanisms of SAL to induce mitochondrial biogenesis. SAL inhibited transcription factors NF-*κ*B activity, enhanced eNOS activity, and NO production, which induced mitochondrial biogenesis subsequently.

In summary, the present study demonstrated a novel mechanism of SAL to protect the endothelial cells from oxidative damage. By stimulating mitochondrial biogenesis (Figure 8) and counteracting the reactive oxygen species burst within mitochondria, SAL administration prevented the overactivation of several signaling pathways evoked by damaging oxidative stimuli and preserved the viability of endothelial cells, as well as the endothelial dependent vessel functions. Novel compounds with mitochondria biogenesis stimulating activities may become potential drug candidates for the prevention or treatment of such disorders associated with oxidative stress as metabolic syndrome or cancer and so forth.

## References

[1] Makarenko VV, Usatyuk PV, Yuan G (2014). Intermittent hypoxia-induced Endothelial barrier Dysfunction requires ROS-dependent MAP kinase activation. *American Journal of Physiology Cell Physiology*.

[2] Hermida N, Balligand JL (2013). Low-density lipoprotein-cholesterol-induced endothelial dysfunction and oxidative stress: the role of statins. *Antioxidants & Redox Signaling*.

[3] Wang S, Zhang M, Liang B (2010). AMPK*α*2 deletion causes aberrant expression and activation of NAD(P)H oxidase and consequent endothelial dysfunction in vivo: role of 26S proteasomes. *Circulation Research*.

[4] Sena CM, Pereira AM, Seica R (2013). Endothelial dysfunction—a major mediator of diabetic vascular disease. *Biochimica et Biophysica Acta*.

[5] Moens AL, Kietadisorn R, Lin JY, Kass D (2011). Targeting endothelial and myocardial dysfunction with tetrahydrobiopterin. *Journal of Molecular and Cellular Cardiology*.

[6] Dromparis P, Michelakis ED (2013). Mitochondria in vascular health and disease. *Annual Review of Physiology*.

[7] Marzetti E, Csiszar A, Dutta D, Balagopal G, Calvani R, Leeuwenburgh C (2013). Role of mitochondrial dysfunction and altered autophagy in cardiovascular aging and disease: from mechanisms to therapeutics. *American Journal of Physiology—Heart and Circulatory Physiology*.

[8] Mercer JR (2014). Mitochondrial bioenergetics and therapeutic intervention in cardiovascular disease. *Pharmacology & Therapeutics*.

[9] Poyton RO, Ball KA, Castello PR (2009). Mitochondrial generation of free radicals and hypoxic signaling. *Trends in Endocrinology and Metabolism*.

[10] Thomas SR, Witting PK, Drummond GR (2008). Redox control of endothelial function and dysfunction: molecular mechanisms and therapeutic opportunities. *Antioxidants & Redox Signaling*.

[11] Sorbara MT, Girardin SE (2011). Mitochondrial ROS fuel the inflammasome. *Cell Research*.

[12] Banfi C, Brioschi M, Barbieri SS (2009). Mitochondrial reactive oxygen species: a common pathway for PAR1- and PAR2-mediated tissue factor induction in human endothelial cells. *Journal of Thrombosis and Haemostasis*.

[13] Ungvari Z, Orosz Z, Labinskyy N (2007). Increased mitochondrial H_2_O_2_ production promotes endothelial NF-*κ*B activation in aged rat arteries. *American Journal of Physiology—Heart and Circulatory Physiology*.

[14] Addabbo F, Ratliff B, Park H-C (2009). The krebs cycle and mitochondrial mass are early victims of endothelial dysfunction: proteomic approach. *American Journal of Pathology*.

[15] Davidson SM, Duchen MR (2007). Endothelial mitochondria: contributing to vascular function and disease. *Circulation Research*.

[16] Kluge MA, Fetterman JL, Vita JA (2013). Mitochondria and endothelial function. *Circulation Research*.

[17] Krishnan KJ, Reeve AK, Samuels DC (2008). What causes mitochondrial DNA deletions in human cells?. *Nature Genetics*.

[18] Rosca MG, Hoppel CL (2010). Mitochondria in heart failure. *Cardiovascular Research*.

[19] Halestrap AP (2006). Calcium, mitochondria and reperfusion injury: a pore way to die. *Biochemical Society Transactions*.

[20] Gutierrez J, Ballinger SW, Darley-Usmar VM, Landar A (2006). Free radicals, mitochondria, and oxidized lipids: the emerging role in signal transduction in vascular cells. *Circulation Research*.

[21] Ungvari Z, Labinskyy N, Gupte S, Chander PN, Edwards JG, Csiszar A (2008). Dysregulation of mitochondrial biogenesis in vascular endothelial and smooth muscle cells of aged rats. *American Journal of Physiology—Heart and Circulatory Physiology*.

[22] Blake R, Trounce IA (2014). Mitochondrial dysfunction and complications associated with diabetes. *Biochimica et Biophysica Acta*.

[23] Scarpulla RC (2011). Metabolic control of mitochondrial biogenesis through the PGC-1 family regulatory network. *Biochimica et Biophysica Acta*.

[24] Patten IS, Arany Z (2012). PGC-1 coactivators in the cardiovascular system. *Trends in Endocrinology and Metabolism*.

[25] Scarpulla RC (2008). Nuclear control of respiratory chain expression by nuclear respiratory factors and PGC-1-related coactivator. *Annals of the New York Academy of Sciences*.

[26] Santos JM, Tewari S, Goldberg AFX, Kowluru RA (2011). Mitochondrial biogenesis and the development of diabetic retinopathy. *Free Radical Biology and Medicine*.

[27] Xu Q, Xia P, Li X, Wang W, Liu Z, Gao X (2014). Tetramethylpyrazine ameliorates high glucose-induced endothelial dysfunction by increasing mitochondrial biogenesis. *PLoS ONE*.

[28] Panossian A, Wagner H (2005). Stimulating effect of adaptogens: an overview with particular reference to their efficacy following single dose administration. *Phytotherapy Research*.

[29] Qian EW, Ge DT, Kong S-K (2011). Salidroside promotes erythropoiesis and protects erythroblasts against oxidative stress by up-regulating glutathione peroxidase and thioredoxin. *Journal of Ethnopharmacology*.

[30] Li X, Sipple J, Pang Q, Du W (2012). Salidroside stimulates DNA repair enzyme Parp-1 activity in mouse HSC maintenance. *Blood*.

[31] Leung SB, Zhang H, Lau CW, Huang Y, Lin Z (2013). Salidroside improves homocysteine-induced endothelial dysfunction by reducing oxidative stress. *Evidence-Based Complementary and Alternative Medicine*.

[32] Williams JR (2008). The declaration of Helsinki and public health. *Bulletin of the World Health Organization*.

[33] Jaffe EA, Nachman RL, Becker CG, Minick CR (1973). Culture of human endothelial cells derived from umbilical veins. Identification by morphologic and immunologic criteria. *The Journal of Clinical Investigation*.

[34] Bai Y, Suzuki AK, Sagai M (2001). The cytotoxic effects of diesel exhaust particles on human pulmonary artery endothelial cells in vitro: role of active oxygen species. *Free Radical Biology and Medicine*.

[35] Song S, Zhou F, Chen WR, Xing D (2013). PDT-induced HSP70 externalization up-regulates NO production via TLR2 signal pathway in macrophages. *FEBS Letters*.

[36] Dasuri K, Ebenezer P, Fernandez-Kim SO (2013). Role of physiological levels of 4-hydroxynonenal on adipocyte biology: implications for obesity and metabolic syndrome. *Free Radical Research*.

[37] Jin S, Lu D, Ye S (2005). A simplified probe preparation for ELISA-based NF-*κ*B activity assay. *Journal of Biochemical and Biophysical Methods*.

[38] Zhang Y, Yang X, Bian F (2014). TNF-*α* promotes early atherosclerosis by increasing transcytosis of LDL across endothelial cells: crosstalk between NF-*κ*B and PPAR-*γ*. *Journal of Molecular and Cellular Cardiology*.

[39] Wang L, Zhen H, Yao W (2011). Lipid raft-dependent activation of dual oxidase 1/H_2_O_2_/NF-*κ*B pathway in bronchial epithelial cells. *American Journal of Physiology—Cell Physiology*.

[40] Zhou R, Yazdi AS, Menu P, Tschopp J (2011). A role for mitochondria in NLRP3 inflammasome activation. *Nature*.

[41] Parra V, Eisner V, Chiong M (2008). Changes in mitochondrial dynamics during ceramide-induced cardiomyocyte early apoptosis. *Cardiovascular Research*.

[42] Reers M, Smith TW, Chen LB (1991). J-aggregate formation of a carbocyanine as a quantitative fluorescent indicator of membrane potential. *Biochemistry*.

[43] Collins T (1993). Biology of disease: endothelial nuclear factor-*κ*B and the initiation of the atherosclerotic lesion. *Laboratory Investigation*.

[44] Schulz E, Gori T, Münzel T (2011). Oxidative stress and endothelial dysfunction in hypertension. *Hypertension Research*.

[45] Giacco F, Brownlee M (2010). Oxidative stress and diabetic complications. *Circulation Research*.

[46] Briasoulis A, Tousoulis D, Androulakis ES, Papageorgiou N, Latsios G, Stefanadis C (2012). Endothelial dysfunction and atherosclerosis: focus on novel therapeutic approaches. *Recent Patents on Cardiovascular Drug Discovery*.

[47] Xu MC, Shi HM, Wang H, Gao XF (2013). Salidroside protects against hydrogen peroxide-induced injury in HUVECs via the regulation of REDD1 and mTOR activation. *Molecular Medicine Reports*.

[48] Yung LM, Leung FP, Yao X, Chen Z-Y, Huang Y (2006). Reactive oxygen species in vascular wall. *Cardiovascular and Hematological Disorders—Drug Targets*.

[49] Félétou M, Vanhoutte PM (2006). Endothelial dysfunction: a multifaceted disorder. *American Journal of Physiology—Heart and Circulatory Physiology*.

[50] Fujimoto S, Asano T, Sakai M (2001). Mechanisms of hydrogen peroxide-induced relaxation in rabbit mesenteric small artery. *European Journal of Pharmacology*.

[51] Yang Z-W, Zhang A, Altura BT, Altura BM (1999). Hydrogen peroxide-induced endothelium-dependent relaxation of rat aorta: involvement of Ca^2+^ and other cellular metabolites. *General Pharmacology*.

[52] Ellis A, Pannirselvam M, Anderson TJ, Triggle CR (2003). Catalase has negligible inhibitory effects on endothelium-dependent relaxations in mouse isolated aorta and small mesenteric artery. *British Journal of Pharmacology*.

[53] Zembowicz A, Hatchett RJ, Jakubowski AM, Gryglewski RJ (1993). Involvement of nitric oxide in the endothelium-dependent relaxation induced by hydrogen peroxide in the rabbit aorta. *British Journal of Pharmacology*.

[54] Cai H, Li Z, Dikalov S (2002). NAD(P)H oxidase-derived hydrogen peroxide mediates endothelial nitric oxide production in response to angiotensin II. *The Journal of Biological Chemistry*.

[55] Wong CM, Yung LM, Leung FP (2008). Raloxifene protects endothelial cell function against oxidative stress. *British Journal of Pharmacology*.

[56] Dimmeler S, Zeiher AM (1999). Nitric oxide—an endothelial cell survival factor. *Cell Death and Differentiation*.

[57] Wong WT, Wong SL, Tian XY, Huang Y (2010). Endothelial dysfunction: the common consequence in diabetes and hypertension. *Journal of Cardiovascular Pharmacology*.

[58] Niu X-F, Smith CW, Kubes P (1994). Intracellular oxidative stress induced by nitric oxide synthesis inhibition increases endothelial cell adhesion to neutrophils. *Circulation Research*.

[59] Cai H, Li Z, Davis ME, Kanner W, Harrison DG, Dudley SC (2003). Akt-dependent phosphorylation of serine 1179 and mitogen-activated protein kinase kinase/extracellular signal-regulated kinase 1/2 cooperatively mediate activation of the endothelial nitric-oxide synthase by hydrogen peroxide. *Molecular Pharmacology*.

[60] Xia Z, Liu M, Wu Y (2006). N-acetylcysteine attenuates TNF-*α*-induced human vascular endothelial cell apoptosis and restores eNOS expression. *European Journal of Pharmacology*.

[61] Mohan S, Hamuro M, Sorescu GP (2003). I*κ*B*α*-dependent regulation of low-shear flow-induced NF-*κ*B activity: role of nitric oxide. *American Journal of Physiology—Cell Physiology*.

[62] Matthews JR, Botting CH, Panico M, Morris HR, Hay RT (1996). Inhibition of NF-*κ*B DNA binding by nitric oxide. *Nucleic Acids Research*.

[63] Pineda-Molina E, Klatt P, Vázquez J (2001). Glutathionylation of the p50 subunit of NF-*κ*B: a mechanism for redox-induced inhibition of DNA binding. *Biochemistry*.

[64] Jornot L, Petersen H, Junod AF (1997). Modulation of the DNA binding activity of transcription factors CREP, NF*κ*B and HSF by H_2_O_2_ and TNF*α*. Differences between in vivo and in vitro effects. *FEBS Letters*.

[65] Oliveira-Marques V, Marinho HS, Cyrne L, Antunes F (2009). Role of hydrogen peroxide in NF-*κ*B activation: from inducer to modulator. *Antioxidants & Redox Signaling*.

[66] Hasegawa Y, Saito T, Ogihara T (2012). Blockade of the nuclear factor-*κ*B pathway in the endothelium prevents insulin resistance and prolongs life spans. *Circulation*.

[67] Nisoli E, Carruba MO (2006). Nitric oxide and mitochondrial biogenesis. *Journal of Cell Science*.

[68] Nisoli E, Clementi E, Paolucci C (2003). Mitochondrial biogenesis in mammals: the role of endogenous nitric oxide. *Science*.

[69] Álvarez-Guardia D, Palomer X, Coll T (2010). The p65 subunit of NF-B binds to PGC-1, linking inflammation and metabolic disturbances in cardiac cells. *Cardiovascular Research*.

[70] Kim H-J, Park K-G, Yoo E-K (2007). Effects of PGC-1*α* on TNF-*α*-induced MCP-1 and VCAM-1 expression and NF-*κ*B activation in human aortic smooth muscle and endothelial cells. *Antioxidants & Redox Signaling*.

[71] Csiszar A, Labinskyy N, Pinto JT (2009). Resveratrol induces mitochondrial biogenesis in endothelial cells. *American Journal of Physiology—Heart and Circulatory Physiology*.

[72] Schilling J, Lai L, Sambandam N, Dey CE, Leone TC, Kelly DP (2011). Toll-like receptor-mediated inflammatory signaling reprograms cardiac energy metabolism by repressing peroxisome proliferator-activated receptor *γ* coactivator-1 signaling. *Circulation: Heart Failure*.

[73] Won JC, Park J-Y, Kim YM (2010). Peroxisome proliferator-activated receptor-*γ* coactivator 1-*α* overexpression prevents endothelial apoptosis by increasing ATP/ADP translocase activity. *Arteriosclerosis, Thrombosis, and Vascular Biology*.

[74] Valle I, Álvarez-Barrientos A, Arza E, Lamas S, Monsalve M (2005). PGC-1*α* regulates the mitochondrial antioxidant defense system in vascular endothelial cells. *Cardiovascular Research*.

[75] Ali F, Ali NS, Bauer A (2010). PPAR*σ* and PGC1*α* act cooperatively to induce haem oxygenase-1 and enhance vascular endothelial cell resistance to stress. *Cardiovascular Research*.

[76] Cai H (2005). Hydrogen peroxide regulation of endothelial function: origins, mechanisms, and consequences. *Cardiovascular Research*.

[77] Liu Y-M, Jiang B, Bao Y-M, An L-J (2008). Protocatechuic acid inhibits apoptosis by mitochondrial dysfunction in rotenone-induced PC12 cells. *Toxicology in Vitro*.

[78] Huang H, Manton KG (2004). The role of oxidative damage in mitochondria during aging: a review. *Frontiers in Bioscience*.

[79] Yakes FM, van Houten B (1997). Mitochondrial DNA damage is more extensive and persists longer than nuclear DNA damage in human cells following oxidative stress. *Proceedings of the National Academy of Sciences of the United States of America*.

[80] Hill BG, Benavides GA, Lancaster JR (2012). Integration of cellular bioenergetics with mitochondrial quality control and autophagy. *Biological Chemistry*.

